# Redesigning and teaching veterinary microbiology laboratory exercises with combined on-site and online participation during the COVID-19 pandemic

**DOI:** 10.1093/femsle/fnab108

**Published:** 2021-08-19

**Authors:** Joanna Koort, Silja Åvall-Jääskeläinen

**Affiliations:** Department of Veterinary Biosciences, Faculty of Veterinary Medicine, University of Helsinki, FIN-00014 Helsinki, Finland; Department of Veterinary Biosciences, Faculty of Veterinary Medicine, University of Helsinki, FIN-00014 Helsinki, Finland

**Keywords:** microbiology, laboratory teaching, online participation, on-site participation, COVID-19, remote partner

## Abstract

The COVID-19 (coronavirus disease 2019) pandemic has forced universities to find new ways to conduct learning and teaching, as traditional face-to-face teaching has been prevented or restricted to an absolute minimum in many instances. Therefore, we redesigned and taught second-year veterinary student microbiology laboratory exercises (labs) with a hybrid learning approach. For this, a novel ‘remote partner’ model was implemented in which students present on-site in the laboratory worked synchronously pairwise with their remote partner present online. A student feedback survey revealed that in this remote partner model, both on-site and online participation in the labs were experienced as being useful in improving their laboratory skills. The students' overall performance in hands-on microbiological laboratory skills and safe working practices was similar in the hybrid learning approach (the 2021 class) and in the traditional on-site participation approach (the 2018–20 classes). This study suggests that the remote partner model is an effective way to acquire microbiological laboratory skills. This learning approach can be used in the non-pandemic future and/or also be applied to other fields.

## Teaser

Microbiological laboratory skills can be developed using a hybrid model combining synchronous on-site and online learning for students working in pairs.

## INTRODUCTION

The University of Helsinki (UH) is Finland's largest academic institution with 11 faculties and 31 600 students. The Faculty of Veterinary Medicine (FVM) of the UH is the only institution educating veterinarians in Finland. Due to the global pandemic of coronavirus disease 2019 (COVID-19), teaching at the UH, including the FVM, was changed mainly to remote teaching after March 2020. Teaching mostly by remote means continued through the autumn 2020 and spring 2021 terms. Only necessary teaching could be conducted as contact teaching on-site in the FVM, and permission for this had to be obtained from the Response Centre of the FVM.

Previous studies have suggested that various online digital tools can be used to assist or even replace laboratory-situated microbiological learning. These tools include, for example, virtual laboratory simulations or exercises (Sancho *et al*. 2006; Salter and Gardner 2016; Brockman *et al*. 2020) and instructional videos (Lacey and Wall 2021). Even though these tools have several advantages, including flexibility in timing and ability to use resources that are difficult to implement on-site (Sancho *et al*. 2006; Brockman *et al*. 2020; Lacey and Wall 2021), none of the online tools can provide hands-on experience similar to exercises held on-site in the microbiological laboratory. Therefore, hands-on laboratory competencies cannot be fully developed by only online teaching tools—personal on-site laboratory practice is also required (Noel *et al*. 2020). Microbiology educators consider that the hands-on laboratory skills that should be taught on-site include safe working practices and manipulation of microbes (Horak 2020). As seen in a recent study, hands-on microbiology laboratory activities were appreciated by medical students (Brockman *et al*. 2020) and this is also likely to be true for veterinary students. Veterinary students also feel that it is difficult to obtain the required veterinary competencies in full with only an online education system (Mahdy 2020). Online teaching may indeed be most useful when it is combined with face-to-face teaching in a hybrid learning model, such as online practical sessions as a precursor to the laboratory exercises (labs) (Salter and Gardner 2016) or alternating online and on-site laboratory sessions (Sancho *et al*. 2006). For these reasons, we decided that the ‘Infection Microbiology’ course labs would include face-to-face on-site teaching in addition to the online teaching even during the COVID-19 pandemic.

‘Infection Microbiology: Bacteriology and Mycology’ is a four-credit compulsory course held once a year (from December to March) for second-year students studying for their Bachelor of Veterinary Medicine degree in the FVM. The course includes lectures and labs, as well as group work and workshops. In 2020–21, the labs were redesigned with a hybrid learning approach and most of the labs were implemented with a ‘remote partner’ model. In this model, students present on-site in the laboratory worked synchronously pairwise with their online remote partner. To ensure the safety of the students and personnel, numerous safety precautions were put into operation during the labs. After the course, students were surveyed about their experiences of the blended on-site and online learning. The achievement of learning outcomes associated with laboratory skills and safe working practices was compared with that obtained with the traditional on-site laboratory learning held before the pandemic. In this article, we describe the redesign and set-up of ‘Infection Microbiology’ labs held during the COVID-19 pandemic. The results of the student survey and the educational outcomes are also reported.

## MATERIALS AND METHODS

### Course set-up

In the academic year 2020–21, 67 students enrolled in the ‘Infection Microbiology’ course. The course started on 8 December 2020 and lasted until 1 March 2021. The main activities were online lectures (21 h), three online group work/workshops, the SafeLab online self-study module and combined on-site/online labs (30 h per student, held over 5 weeks in January–February 2021). The SafeLab module and labs were compulsory, while most of the other exercises were voluntary. The learning outcomes of the ‘Infection Microbiology’ course are shown in Table 1. Assessment of the learning outcomes was based on two obligatory exams—a practical exam held during the last week of the labs and a written final exam held remotely—as well as on accepted performance in all the compulsory activities.

**Table 1.** Learning outcomes of ‘Infection Microbiology’ course. Outcomes related to labs are marked in bold.

Student:
• **is familiar with veterinary important infectious bacteria and fungi, the main diseases they cause and their main pathogenic properties**.
• **can work in a microbiological laboratory safely and aseptically**.
• **can select and perform basic bacteriological diagnostic methods using information sources**.
• understands the differences between the spectra of different antimicrobial groups and drugs belonging to these groups, for bacteria at both the group and genus levels.
• **understands the factors influencing the results and reliability of bacteriological tests** and antimicrobial susceptibility tests, **andcan apply information sources in the interpretation of test results**.

All the course materials were available via the UH's Moodle learning management system (https://moodle.org/). In addition to lecture notes, the Moodle course area also had voluntary material supporting the achievement of learning outcomes, such as interactive exercises, weblinks, scientific articles and links to e-books.

### Structure of the labs

Because ‘Infection Microbiology’ is the first practical microbiological course for the students, each student is required to pass a SafeLab online self-study module prior to attending the labs. This module consists of Moodle lessons and two online tests related to safe working and good operating practices in the microbiology laboratory and the basic methods used in the course.

The labs are divided into five laboratory weeks (Table 2). The FVM has two microbiological teaching laboratories located next to each other. During the labs, one teacher and one teaching assistant guide students in each laboratory. The students enrolled in the course are arranged into groups 1–3, which in 2021 included 24, 26 and 17 students, respectively. Most of the exercises are done in pairs. Students may choose their laboratory partner themselves; if they do not want to make the choice themselves, the teacher makes this by drawing lots.

**Table 2.** Laboratory activities included in the ‘Infection Microbiology’ course.

Activity	Description	Exercise format
**Lab week 1** Introduction to practical bacteriological laboratory work	2 × 2 h of labs with the aim of getting acquainted with basic methods and working principles used during the course: streak plate method, Gram staining, light microscopy, aseptic techniques	Individual on-site participation
**Lab week 2** Identifying an unknown bacterium (Gram-positive coccus)	3 × 2 h of labs with Gram-positive cocci	Pairs online or on-site participation
**Lab week 3** Identifying an unknown bacterium (Gram-positive rod)	3 × 2 h of labs with Gram-positive rods	Pairs online or on-site participation
**Lab week 4** Identifying an unknown bacterium (Gram-negative rod)	4 × 2 h of labs with Gram-negative rods	Pairs online or on-site participation
**Lab week 5** Practical exam identifying an unknown bacterium (Gram-positive/negative, coccus/rod)	3 × 2 h of labs with each laboratory pair working to solve the identity of a bacterium given to them by code name only	Pairs on-site participation

Each laboratory session begins with a teacher-led 10–15-min introduction to the day's work. After the introduction, students work independently with the help of written work instructions and other study material available as PDF files on Moodle. The main theme of each laboratory week is to identify a given unknown bacterial strain or strains with an anamnesis, that is patient case history, related to each strain. For this, basic bacteriological methods such as Gram staining and light microscopy, culturing of bacteria in various growth media and basic biochemical assays are practised by the students. The final day of the laboratory week ends with a teacher-led summary lecture during which results obtained by students are also considered. In 2021, the introduction and summary lectures were presented online using Zoom.

### Redesigning laboratory-based teaching methods with hybrid learning approach

As institutional policy mandated that the maximum number of people present in the same room during on-site teaching was 20 and social distancing (1–2 m) had to be observed whenever possible, laboratory modifications were needed. The main pedagogical modification was to combine on-site and online participation in the labs. For this, a ‘remote partner’ model was designed. In this model, students work in pairs so that one is present in the laboratory as an on-site partner, while the other member participates online as a remote partner. This model was selected because the number of teacher contact hours could not be increased and there was no willingness to reduce the number of student contact hours.

The laboratory activities included in ‘Infection Microbiology’ are shown in Table 2. To ensure that all the students achieved similar basic-level practical knowledge of laboratory safety and basic microbiological methods, all students participated on-site and worked individually in Lab week 1. Only one group was present at a time, and the group was divided between the two adjacent laboratories.

During Lab weeks 2–4 students worked in pairs, and the remote partner model was in use. The laboratory pairs communicated pairwise via Zoom. The on-site partner used Zoom either via an iPad made available in the laboratory for each student by the FVM or via a student's own smartphone. The on-site partner performed the practical parts of the exercise in the laboratory, while the remote partner was instructed to focus on supervision of the work, gathering information and forming conclusions. For each session, pairs could decide themselves which of them participated on-site and which of them participated as a remote partner. Even though there was no minimum on-site laboratory hour requirement, students were advised to share the on-site and remote roles as equally as was feasible. To ensure that all the students participated on-site sufficiently to get a basic level of practical knowledge, each student was mandated to perform at least a certain number of Gram stains and streak plates in the laboratory during these weeks. The students filled out a form about these tasks. The teachers made sure that each student completed these tasks.

During the practical exam held in Lab week 5, students worked in pairs so that both partners were simultaneously present in the labs but sat at adjacent workstations. As in Lab week 1, only one group was present at a time, and the group was divided between the two adjacent laboratories.

### Biosafety modifications of the labs during the COVID-19 pandemic

With these pedagogical arrangements, the number of people (including teachers) per laboratory varied from 9 to 16, depending on the group and laboratory week, and was thus well below the maximum permitted. Students were advised to be prepared to change their scheduled on-site/online roles at short notice before laboratory sessions. Teachers were also prepared to rearrange student pairs or groups if both students in a pair or a larger number of students were quarantined. Participation in the labs was strictly prohibited for people suspected of having the COVID-19 infection. Students were not tested for COVID-19 by the FVM but taking a COVID-19 test was strongly encouraged for anyone with symptoms.

During the labs, students and teaching personnel wore masks. Numerous other COVID-19 related biosafety measures (Table 3) were also applied. These measures were targeted to minimize the risk of COVID-19 transmission by (i) ensuring that social distancing was realized as much as possible or (ii) disinfecting the surfaces.

**Table 3.** Safety actions modified due to COVID-19 pandemic and implemented during labs.

Action	Further description	Difference(s) related to labs held before COVID-19 pandemic
Entry and exit to the laboratory	Laboratory doors wedged open	Doors kept closed
Seating places	Predefined seating places	Students could decide themselves their seating place (same throughout the course)
Distribution of materials to students	Materials needed during the labs (e.g. growth media, iPads) provided directly to each student's workstation or shelf on the island	Materials needed during the labs were provided at a single place in the laboratory and collected by the students
Placing of light microscopes	Microscopes were placed in the laboratory with COVID-19 safety distances	Microscopes were located close to each other
Use of light microscopes	Each student used the microscope located closest to her/him. Microscopes were located so that cross-movement between students sitting on different islands would not happen. After using the microscope, student disinfected all the microscope surfaces she/he touched by using a disinfectant wipe. This protocol was also employed on ocular surfaces when teacher came to look/advise the students about their staining or help with use of microscope. Students waited for their microscopy turn at their own workstation	Students could use any available microscope in the laboratory. Microscopes were not disinfected after use (only normal cleaning of objectives was performed). Student would go to microscopes and wait for their turn there
Use of fume cupboards	Students went to the fume cupboard only when there was no other student working there	Students would line up in front of the fume cupboards to wait for their turn
Bacterial cultures made by the students during the labs	Each student's workstation table was marked with taped signs indicating a place for cultures going to either +37°C or +4°C. After the labs ended, teacher took the cultures to the temperatures indicated by the student	Students took all their cultures to/from the incubator or refrigerator themselves
Demonstration materials (e.g. ready-made microscope preparates)	Demonstration materials were common to students sitting on the same island	Demonstration materials were common to all students in the group
Disinfection of surfaces at the end of each laboratory session	Students disinfected all the surfaces and objects at their workstation that they had touched during the labs	Students routinely disinfected only the table of their workstation (plus any accidentally contaminated surfaces)
Washing and disinfecting hands during the labs	Students waited for their turn and used the nearest hand basin	Students would line up in front of the hand basins to wait for their turn
Laboratory coat racks	Laboratory coat racks were marked pair- and groupwise	Students could leave their laboratory coats on any rack available

To ensure social distancing, student seats were prearranged by the teachers (Fig. S1, Supporting Information). All the labs were designed so that students could stay seated at their own workstation and avoid moving around the laboratory as much as possible. Special attention was paid to avoid cross-movement between students sitting on different islands.

### Evaluation of the remote partner model

The influence of the remote partner model on learning was evaluated in two different ways: evaluating student performance and collecting student feedback.

The teachers evaluated student performance in the hands-on microbiological laboratory skills by conducting a practical exam in Lab week 5. In the practical exam, students working in pairs identified an unknown bacterium. Performance was assessed using grades 0–3 across each of the following seven tasks/task groups: ‘Listing of the potential differential pathogens on the grounds of a given anamnesis’, ‘Gram stain’, ‘Streak culture’, ‘Describing the colony morphology and recognizing the possible impurity of the culture’, ‘Selecting and performing necessary microbiological tests’, ‘Making the bacterial diagnosis based on the results obtained’ and ‘Determining the significance of an identified bacterium as the cause of the case’. The task ‘Safety’ was assessed with 2-fold weight and was thus graded 0–6. The results were compared with the results from previous years (2018–20) when only traditional on-site based laboratory learning was in use but the practical exam was similar to that held in 2021.

After the written exam, students were invited via Moodle to answer a web-based feedback survey. The response time was 12 days, and participation was both voluntary and anonymous. No personal information was collected. The questionnaire included 14 propositions/proposition groups with supplemental open fields and four open-ended questions. Eight of the propositions/proposition groups were used in this study (Table S1, Supporting Information). Three of these, namely ‘Overall grade to the course’, ‘Workload of the course was in relation to the number of course credits obtained’ and ‘Laboratory exercises helped me to understand the course subject’, had also been used in the previous years and were thus used in comparisons between 2021 and years 2018–20. Student experiences of the remote partner model were sought with several propositions (Table S1, Supporting Information). To enable comparison between on-site and remote partner aspects, the skill-related questions were asked twice, similarly for both aspects.

For most of the proposition answers, a five-point Likert scale was used (1 = strongly disagree, 5 = strongly agree). Exceptions were the proposition ‘Workload of the course was in relation to the number of course credits obtained’ in 2018, which was thus excluded from the analyses, and a proposition concerned with network connection problems in 2021, in which a three-point frequency scale (1 = >3 times, 2 = 1–3 times, 3 = never) was used.

Analyses were performed with IBM SPSS Statistics 27 (IBM, New York, USA) for Windows. The Mann–Whitney U test was used for comparisons, as the variables were not normally distributed. Statistical significance was set at *P *= 0.05.

## RESULTS

### Student performance

Sixty-seven students participated in the practical exam in 2021 and 200 in 2018–20. Student performance in the practical exam in 2021 was similar to the previous years (2018–20) when the distributions of the total scores for the exam were compared. However, several statistically significant (*P *≤ 0.048) differences were seen when the distributions of scores for individual tasks were compared (Table 4). The means of five tasks/task groups, namely ‘Listing of the potential differential pathogens on the grounds of a given anamnesis’, ‘Streak culture’, ‘Selecting and performing necessary microbiological tests’, ‘Making the bacterial diagnosis based on the results obtained’ and ‘Determining the significance of an identified bacterium as the cause of the case’, were lower in 2021 than in the previous years. However, the differences were small (0.079–0.221 points). Interestingly, the mean of the task ‘Describing the colony morphology and recognizing the possible impurity of the culture’ was higher (0.503 points) than in the previous years.

**Table 4.** Comparisons between 2021 and 2018–20 by practical exams and surveys. Grading scale, if other than 0–3, is specified in the table. Significant *P*-values (<0.050) are marked in bold.

Task or proposition		Category (*n*)	Mean (95% CI^a^)	Min.^b^	Max.^b^	Mann–Whitney U test *P*-value
Practical exam	Safety (grading scale: 0–6)	2021 (*n* = 67)	5.015 (4.687–5.342)	2.0	6.0	0.054
		2018–20 (*n* = 200)	5.217 (5.035–5.399)	0.0	6.0	
	Listing potential differential pathogens	2021 (*n* = 67)	1.985 (1.814–2.156)	0.0	3.0	**0.048**
		2018–20 (*n* = 200)	2.196 (2.097–2.097)	0.0	3.0	
	Gram stain	2021 (*n* = 67)	2.455 (2.338–2.573)	1.5	3.0	0.160
		2018–20 (*n* = 200)	2.362 (2.290–2.435)	0.9	3.0	
	Streak culture	2021 (*n* = 67)	2.045 (1.882–2.207)	1.0	3.0	**0.005**
		2018–20 (*n* = 200)	2.231 (2.168–2.294)	1.3	3.0	
	Describing the colony morphology	2021 (*n* = 67)	2.679 (2.524–2.835)	0.0	3.0	**<0.001**
		2018–20 (*n* = 200)	2.176 (2.059–2.292)	0.0	3.0	
	Selecting and performing the tests	2021 (*n* = 67)	2.373 (2.260–2.486)	1.5	3.0	**0.046**
		2018–20 (*n* = 200)	2.530 (2.471–2.589)	1.0	3.0	
	Bacterial diagnosis	2021 (*n* = 67)	2.642 (2.478–2.806)	0.0	3.0	**<0.001**
		2018–20 (*n* = 200)	2.721 (2.639–2.803)	0.0	3.0	
	Determining the significance of identified bacterium	2021 (*n* = 67)	1.896 (1.769–2.022)	1.5	3.0	**<0.001**
		2018–20 (*n* = 200)	2.117 (2.048–2.185)	0.0	3.0	
	Total points	2021 (*n* = 67)	21.090 (20.499–21.680)	14.0	25.0	0.409
		2018–20 (*n* = 200)	20.814 (20.416–21.212)	13.5	25.9	
Survey	Overall grade in the course	2021 (*n* = 33)	4.33 (4.12–4.54)	3	5	0.787
		2018–20 (*n* = 64)	4.38 (4.24–4.51)	3	5	
	Workload of the course was in relation to the number of course credits	2021 (*n* = 33)	4.03 (3.80–4.26)	2	5	**0.044**
		2019–20 (*n* = 37)	3.70 (3.42–3.98)	1	5	
	Laboratory exercises helped me to understand the course subject	2021 (*n* = 33)	4.76 (4.60–4.91)	4	5	0.859
		2018–20 (*n* = 64)	4.72 (4.56–4.88)	1	5	

^a^CI = confidence interval.

^b^Min./Max. = lowest and highest scores of the task/proposition, respectively.

### Student feedback

A total of 33 students responded to the feedback survey in 2021 and 64 during years 2018–20. Between these year groups, the distribution of answers for propositions ‘Overall grade to the course’ and ‘Laboratory exercises helped me to understand the course subject’ was similar. However, there was a significant difference (*P *= 0.044) in the distribution of answers for the proposition ‘Workload of the course was in relation to the number of course credits obtained’ (Table 4). Surprisingly, in 2021 the mean for this proposition was higher, that is the workload of the course was experienced as being more reasonable than in the previous years.

**Figure 1.**
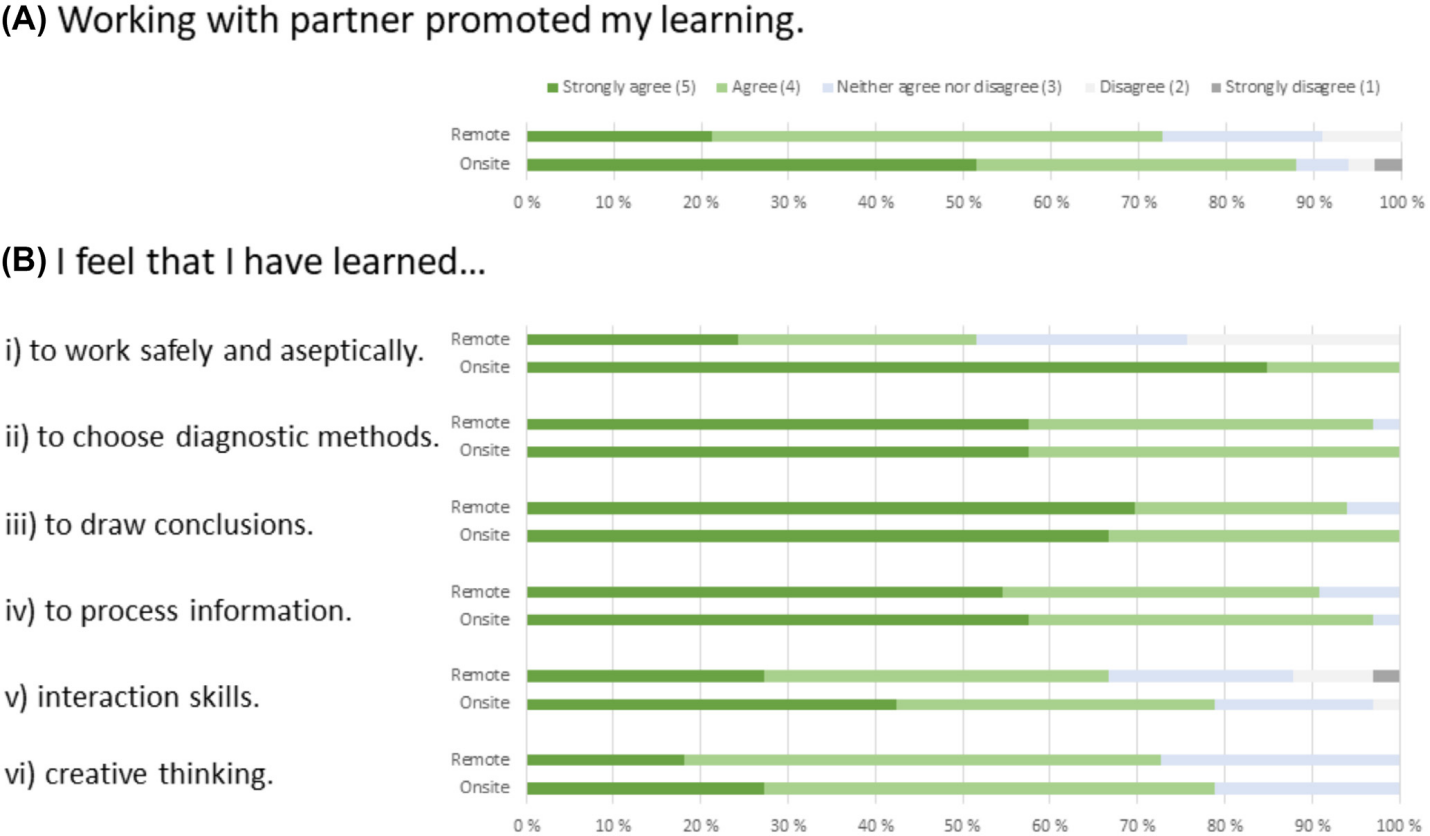
The survey results on the student experiences of the remote partner model. A five-point Likert scale was used in propositions. **(A)** While participating on-site/online, working with my remote/on-site partner promoted my own learning. **(B)** While participating in lab teaching on-site/online, I feel that I have learned (i) to work safely and aseptically in the microbiological laboratory; (ii) to choose basic methods of bacteriological diagnostics by utilizing information sources; (iii) to draw reasonable conclusions from the results I have obtained; (iv) to process information; (v) interaction skills; and (vi) creative thinking.

Student experiences of the remote partner model were mainly positive and similar in both on-site and remote aspects. While most of the students indicated that both on-site and online participation in the labs with a remote/on-site partner improved their learning (Fig. 1A), there were statistically significant (*P *= 0.013) differences between on-site and remote aspects in the distribution of answers (Table 5). However, when the five-point scale was converted into a three-point scale by combining the ‘Strongly agree’ with ‘Agree’ and ‘Strongly disagree’ with ‘Disagree’ answers, the statistical significance disappeared (*P *= 0.141). Almost 90% of the students strongly agreed or agreed that their learning improved because of working on-site in the laboratory with their remote partner. Approximately 70% of the students strongly agreed or agreed that their learning improved because of working remotely online with their on-site partner (Fig. 1A). Analysis of student comments in the supplemental open field suggested that the biggest challenges, especially during remote online participation, were technical issues (background noise, etc.) that could have affected the learning experience. However, students also reported that online participation promoted learning of the theoretical matters related to the labs.

**Table 5.** Comparison between student (*n* = 33) experiences of the on-site and remote aspects in 2021. Response options, if other than five-point Likert scale, are specified in the table. Significant *P*-values (<0.050) are marked in bold.

Proposition	Category	Mean (95% CI^a^)	Min.^b^	Max.^b^	Mann–Whitney U test *P*-value
Did you have problems with the internet connection during the laboratory exercises? Scale: never (3), 1–3 times (2), >3 times (1)	On-site	2.45 (2.28–2.63)	2	3	**<0.001**
	Remote	2.85 (2.72–2.98)	1	3	
While participating on-site/online, working with my remote/on-site partner promoted my own learning	On-site	4.30 (3.97–4.64)	1	5	**0.013**
	Remote	3.85 (3.54–4.16)	2	5	
I learned to work safely and aseptically in the microbiological laboratory	On-site	4.85 (4.72–4.98)	4	5	**<0.001**
	Remote	3.52 (3.12–3.91)	2	5	
I learned to choose basic methods of bacteriological diagnostics by utilizing information sources	On-site	4.58 (4.40–4.75)	4	5	0.917
	Remote	4.55 (4.35–4.75)	3	5	
I learned to draw reasonable conclusions from the results obtained	On-site	4.67 (4.50–4.84)	4	5	0.931
	Remote	4.64 (4.42–4.85)	3	5	
I learned to process information	On-site	4.61 (4.43–4.78)	4	5	0.438
	Remote	4.45 (4.22–4.69)	3	5	
I learned interaction skills	On-site	4.18 (3.88–4.48)	2	5	0.129
	Remote	3.79 (3.42–4.16)	1	5	
I learned creative thinking	On-site	4.06 (3.81–4.31)	3	5	0.374
	Remote	3.91 (3.67–4.15)	3	5	

^a^CI = confidence interval.

^b^Min./Max. = lowest and highest scores of the proposition, respectively.

Overall, experience of the labs, in terms of both on-site and remote aspects, was seen as a useful way to gain laboratory skills and certain general learning skills. All six propositions in this group attracted more positive responses than negative ones (Fig. 1B). Students experienced on-site and remote participation as mostly being equally useful. The only proposition for which there were statistically significant (*P *< 0.001) differences in the distribution of student answers between on-site and remote participation was related to working practices (Table 5). This difference existed even when the five-point scale was converted into a three-point scale (*P *< 0.001). All students strongly agreed or agreed that they learned to work safely and aseptically in the laboratory because of the on-site labs. This was also seen in the student comments in the open field—the ‘trial and error’ was seen as a valuable learning method in on-site labs. In contrast, only about half of the students strongly agreed or agreed that they learned to work safely and aseptically in the laboratory as a result of online participation in the labs (Fig. 1B).

While most of the students (60.6%) experienced occasional internet network problems while participating on-site in the labs, only 15.2% reported these while participating remotely. The difference in the distribution of these answers was statistically significant (*P *< 0.001; Table 5). In the laboratory, most of the students were able to use the iPads excellently (60.6%) or well (27.3%) to support their on-site learning. However, some challenges related to the use of iPads were reported, for example, in file management.

## DISCUSSION

Because of the importance of laboratory-situated microbiological learning, we implemented a novel remote partner model that enabled flexible participation of the students in the labs held on-site during the COVID-19 pandemic. This model combines synchronous on-site and online learning and encourages collaborative student problem-solving. To our knowledge, this kind of hybrid learning model for gaining microbiological laboratory skills has not been reported previously.

Student overall performance in the practical exam, which measures laboratory skills and safe working practices, was similar in the remote partner model (the 2021 class) and in the traditional approach with solely on-site laboratory participation (the 2018–20 classes). Detailed analysis of the results of the individual tasks in the practical exam revealed a higher level of performance in the task ‘Describing the colony morphology and recognizing the possible impurity of the culture’ in 2021 than in the previous years. This may be a result of increased verbal communication between the laboratory partners in the remote partner model. As the visibility of details (e.g. bacterial colony morphology) through the screen may be limited, the partners had to communicate more with each other and explain details orally during the labs. Previously, when both partners worked on-site, the emphasis of the pairwise communication might have been different, for example concentrating more on the distribution of daily tasks between the partners. Working in pairs per se is also beneficial for the students, because when students work together, their understanding of the content is improved (Salter and Gardner 2016).

Our study revealed that students were very satisfied with the remote partner model. Both on-site and remote participation in the labs were regarded as being a useful way to gain laboratory skills and certain general learning skills. However, according to student responses, safe and aseptic working practices were not as easy to learn online as on-site. Even though student experiences in this matter were not reflected in their overall performance in the practical exam, we conclude that a certain amount of laboratory-situated contact learning is needed to develop these practices. This conclusion is supported by Sancho *et al*. (2006), who stated that aseptic techniques must be learned on-site in the real laboratory. Manual repetition of techniques also brings useful long-term muscle memory that cannot be created in online exercises. In the real laboratory, students also often learn through the mistakes they make, which are less easy to forget than they would be in online simulations or exercises.

Somewhat surprisingly, the students experienced the workload of the course in relation to the number of course credits obtained to be more reasonable in 2021 compared with the pre-pandemic years. Studies have shown that online learning can be quite stressful to the students (Mheidly, Fares and Fares 2020; Oducado and Estoque 2021) and our course had substantially more online learning in 2021 than in the previous years. We speculate that one factor influencing student workload experience was the fact that for the participating students this was their first course with regular contact teaching since the beginning of the COVID-19 imposed restrictions. This opportunity to participate on-site with fellow students after a long break probably increased student motivation, thus lightening their experience of the study load.

Most of the students experienced occasional internet network errors while participating on-site in the labs, while few reported these while participating remotely. However, the on-site network problems encountered were temporary and the students were always able to connect with their partner during each class, either by using an alternative internet network source or by simply reconnecting. A good internet connection between the laboratory partners is an absolute requirement for successful synchronous working in the remote partner model.

On the grounds of the student feedback, a few practical changes to the labs are suggested. First, portable stands for the iPads should be provided. Without a stand, it was sometimes challenging for the students to position and especially keep the iPad still at a good camera angle at their workstation. Second, all the students in the laboratory should be strongly encouraged to use earbuds when in contact with their remote partners. Several students reported hearing disturbing background noise in the labs that most likely resulted from the voices of remote partners coming from the iPads’ speakers.

In hindsight, a few changes to the teaching practices are also suggested. Even though the remote partners interacted actively with their on-site laboratory partners during our course, this interaction and the active role of the remote partner could still be enhanced. This could be achieved by preparing some daily laboratory-related questions that the partners should discuss during the labs, but it would be the responsibility of the remote partner to return the answers to the Moodle course area. To assess the effect of the remote partner model on student collaboration and communication skills, learning outcomes relating to these should be included in the course syllabus. To be better prepared for a possible long absence of some students from on-site laboratory teaching, for example due to COVID-19-related quarantine, time for compensatory extracurricular laboratory hours before the practical exam should be scheduled beforehand.

In the future, the remote partner model described here could be applied in two principal ways. For an individual student, the model can be applied in situations that hinder the student's physical/on-site participation. Contrary to, for example, a compensatory written assignment, synchronous online participation allows the student to engage in the labs in multiple ways and be of true help to the on-site partner in gathering information and forming conclusions. For an entire class, as in this study, the remote partner model could also be used whenever a hybrid learning approach is necessary or desirable. In this case, it is important to control that the laboratory partners share their on-site and remote roles equally.

In conclusion, the results of this study suggest that the remote partner model is an effective way to acquire microbiological laboratory skills. This model could also be applied to other fields in which practical skills need to be learned, pairwise working is considered beneficial and some of the students are not able to participate in the contact teaching on-site.

## Supplementary Material

fnab108_Supplement_Files

## References

[bib1] Brockman RM, Taylor JM, Segars LW et al. Student perceptions of online and in-person microbiology laboratory experiences in undergraduate medical education. Med Educ Online. 2020;25:1710324. 31928152 10.1080/10872981.2019.1710324 PMC7006765

[bib2] Horak R. Virtual Resources to Teach Microbiology Techniques and Experiments. 2020. https://asm.org/Articles/2020/December/Virtual-Resources-to-Teach-Microbiology-Techniques (13 July 2021, date last accessed).

[bib3] Lacey K, Wall JG. Video-based learning to enhance teaching of practical microbiology. FEMS Microbiol Lett. 2021;368:fnaa203. 33351097 10.1093/femsle/fnaa203

[bib4] Mahdy MAA. The impact of COVID-19 pandemic on the academic performance of veterinary medical students. Front Vet Sci. 2020;7:594261. 33134368 10.3389/fvets.2020.594261 PMC7572855

[bib5] Mheidly N, Fares MY, Fares J. Coping with stress and burnout associated with telecommunication and online learning. Front Public Health. 2020;8:574969. 33262967 10.3389/fpubh.2020.574969 PMC7686031

[bib6] Noel TC, Rubin JE, Acebo Guerrero Y et al. Keeping the microbiology lab alive: essential microbiology lab skill development in the wake of COVID-19. Can J Microbiol. 2020;66:603–4. 32758098 10.1139/cjm-2020-0373

[bib7] Oducado RMF, Estoque HV. Online learning in nursing education during the COVID-19 pandemic: stress, satisfaction, and academic performance. J Nurs Pract. 2021;4:143–53.

[bib8] Salter S, Gardner C. Online or face-to-face microbiology laboratory sessions? First year higher education student perspectives and preferences. Creat Educ. 2016;7:1869–80.

[bib9] Sancho P, Corral R, González MJ et al. A blended learning experience for teaching microbiology. Am J Pharm Educ. 2006;70:120. 17149449 10.5688/aj7005120 PMC1637024

