# Supplementary material for: Redesigning and teaching veterinary microbiology laboratory exercises with combined on-site and online participation during the COVID-19 pandemic
Source: FEMS Microbiol Lett. 2021 Aug 19;368(16):fnab108. doi: 10.1093/femsle/fnab108 (PMC8390828; doi:10.1093/femsle/fnab108)
Supplement: fnab108_Supplement_Files [file fnab108_supplement_files.zip › Suppl_Table_1.docx]

**Supplementary Table 1.** Student feedback survey propositions used in this study.

| **Proposition** | **Response options†** | **Used in year(s)** |
| --- | --- | --- |
| What is the overall grade you gave to the course? | Excellent (5), Good (4), Satisfactory (3), Poor (2), Very poor (1) | 2018–21 |
| Workload of the course was in relation to the number of course credits obtained. |  | 2019–21 |
| Laboratory exercises helped me to understand the course subject. |  | 2018–21 |
| Did you have problems with the Internet connection during the labs while you were   - 1. onsite?   2. online? | Never (3), 1-3 times (2), More than three times (1) | 2021 |
| How well were you able to utilise the iPads in the lab to support your learning? | Excellently (5), Well (4), Satisfactorily (3), Tolerably (2), Not at all (1) | 2021 |
| While participating…   - 1. …onsite, working with my remote partner promoted my own learning.   2. …online, working with my onsite lab partner promoted my own learning. |  | 2021 |
| While participating in lab teaching…  …onsite, I feel I have learnt:  …online, I feel I have learnt:   - to work safely and aseptically in the microbiological laboratory. - to choose basic methods of bacteriological diagnostics by utilising information sources. - to draw reasonable conclusions from the results I have obtained. - to process information. - interaction skills. - creative thinking. |  | 2021  2021 |

†Response options, if other than Strongly disagree (1), Disagree (2), Neither agree nor disagree (3), Agree (4), Strongly agree (5), are specified in the table.
