# Supplementary material for: Redesigning and teaching veterinary microbiology laboratory exercises with combined on-site and online participation during the COVID-19 pandemic
Source: FEMS Microbiol Lett. 2021 Aug 19;368(16):fnab108. doi: 10.1093/femsle/fnab108 (PMC8390828; doi:10.1093/femsle/fnab108)
Supplement: fnab108_Supplement_Files [file fnab108_supplement_files.zip › supplemental_material.docx]

Supplemental material, intended for eventual online publication as Supporting Information:

Supplemental Figure 1. Floor plan with positioning of relevant furniture and equipment, such as students’ workstations and microscopes.

Supplementary Table 1. Student feedback survey propositions used in this study.
