# Supplementary figures and images for: Redesigning and teaching veterinary microbiology laboratory exercises with combined on-site and online participation during the COVID-19 pandemic
Source: FEMS Microbiol Lett. 2021 Aug 19;368(16):fnab108. doi: 10.1093/femsle/fnab108 (PMC8390828; doi:10.1093/femsle/fnab108)

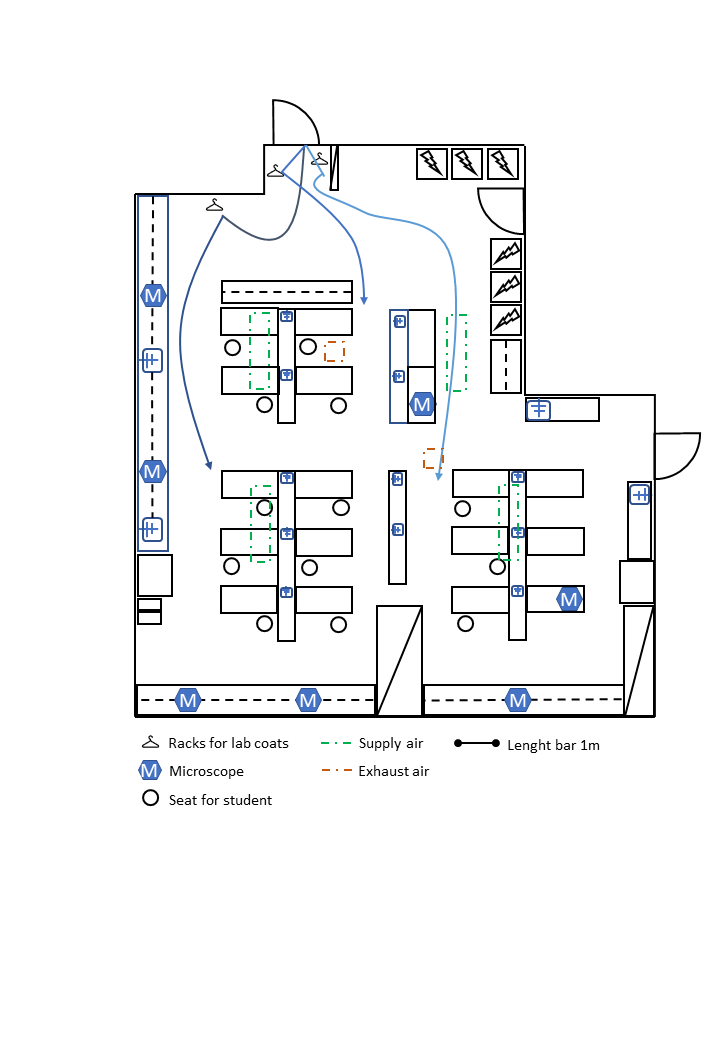

Supplement: fnab108_Supplement_Files [file fnab108_supplement_files.zip › Suppl_Fig_1300.tif]
